# Thrombolysis in Children: A Case Report and Review of the Literature

**DOI:** 10.3389/fped.2021.814033

**Published:** 2022-01-24

**Authors:** Gary M. Woods, Dennis W. Kim, Matthew L. Paden, Heather K. Viamonte

**Affiliations:** Children's Healthcare of Atlanta, Emory University School of Medicine, Atlanta, GA, United States

**Keywords:** thrombosis, thrombolysis, pediatrics, extracorporeal life support (ECLS), COVID-19, May-Thurner, Paget-Schroetter

## Abstract

Thromboembolism (TE), including venous thromboembolism (VTE), arterial TE, arterial ischemic stroke (AIS), and myocardial infarction (MI), is considered a relatively rare complication in the pediatric population. Yet, the incidence is rising, especially in hospitalized children. The vast majority of pediatric TE occurs in the setting of at least one identifiable risk factor. Most recently, acute COVID-19 and multisystem inflammatory syndrome in children (MIS-C) have demonstrated an increased risk for TE development. The mainstay for the management pediatric TE has been anticoagulation. Thrombolytic therapy is employed more frequently in adult patients with ample data supporting its use. The data for thrombolysis in pediatric patients is more limited, but the utilization of this therapy is becoming more commonplace in tertiary care pediatric hospitals. Understanding the data on thrombolysis use in pediatric TE and the involved risks is critical before initiating one of these therapies. In this paper, we present the case of an adolescent male with acute fulminant myocarditis and cardiogenic shock likely secondary to MIS-C requiring extracorporeal life support (ECLS) who developed an extensive thrombus burden that was successfully resolved utilizing four simultaneous catheter-directed thrombolysis (CDT) infusions in addition to a review of the literature on the use of thrombolytic therapy in children.

## Case

A 12-year-old 87.9 kg male presented with five days of fever and two days of midsternal chest pain and respiratory distress. Initial lab evaluations showed a mild leukocytosis with neutrophil predominance, elevated d-dimer (3,954 ng/mL), elevated inflammatory markers (fibrinogen 1,288 mg/dL; lactic acid 3.8 mmol/L; C-reactive protein 26 mg/L; ferritin 379 ng/mL), a troponin of 12.5 ng/mL, and a BNP of 1,740 pg/mL. Influenza and SARS-CoV-2 PCR testing were negative. It was noted that his father worked in a poultry plant with multiple known SARS-CoV-2 positive co-workers. Electrocardiogram showed diffuse ST elevation and a chest radiograph showed bilateral airspace opacities and prominent heart size. He was intubated shortly after arrival due to hemodynamic instability. An echocardiogram revealed severely depressed biventricular systolic function, moderate mitral valve regurgitation, and a small right atrial (RA) thrombus. Due to refractory cardiogenic shock from suspected myocarditis, he was cannulated onto veno-arterial (VA)-ECLS via the right femoral artery with termination in the descending aorta and right femoral vein with termination at the inferior vena cava (IVC) and RA junction.

Unfractionated heparin (UFH) infusion was used for anticoagulation (100 units/kg IV at the time of cannulation, followed by an infusion at 25 units/kg/h), but despite reasonable iSTAT kaolin activated clotting times (ACTs) of 180–200 s, therapeutic heparin assays (0.35–0.7 units/mL) could not be achieved. Significant fibrin deposits in the ECLS circuit developed in the first hour after cannulation and the patient was transitioned to bivalirudin [0.25 mg/kg/h starting dose with escalation up to 1.15 mg/kg/h based on activated partial thromboplastin time (aPTT) goal of 2–3 times his baseline (60–85 s)] due to concern for heparin resistance. One significant limitation to bivalirudin that we discussed was its inability to provide clot dissolution in areas of stagnant flow, for example a poorly contracting ventricle with little inflow/outflow, but the inability to achieve therapeutic heparin assays was deemed a more significant risk.

Four hours post-cannulation, repeat echocardiogram revealed a massive thrombus burden [right ventricular apex thrombus, large right pulmonary artery (RPA) thrombus from the branch bifurcation throughout the mid and distal portions, large left pulmonary artery (LPA) thrombus in the proximal portion, mural thrombus in the left ventricular apex (3.1 cm), and large thrombus in the ascending aorta (3.4 x 1.4 cm) with extension into the transverse arch].

After confirming that the patient had no intracranial abnormalities by non-contrast head computed tomography (CT), systemic thrombolysis with recombinant tissue plasminogen activator (rtPA) (100 mg over 2 h) was initiated via his right internal jugular central venous line. Due to concerns that the extensive nature of the thrombus burden that may not be resolved with a single systemic thrombolysis infusion and his ongoing significant thrombotic risk, this was followed by a continuous rtPA infusion at our institutional maximum dose of 1 mg/h. A bivalirudin infusion was run currently during the rtPA infusions with an aPTT goal of ~1.5–2 times his baseline (50–60 s). Repeat echocardiographic imaging after 12 h revealed little change in thrombotic burden with slight extension of the aortic thrombus farther into the transverse arch.

**Figure 1:**
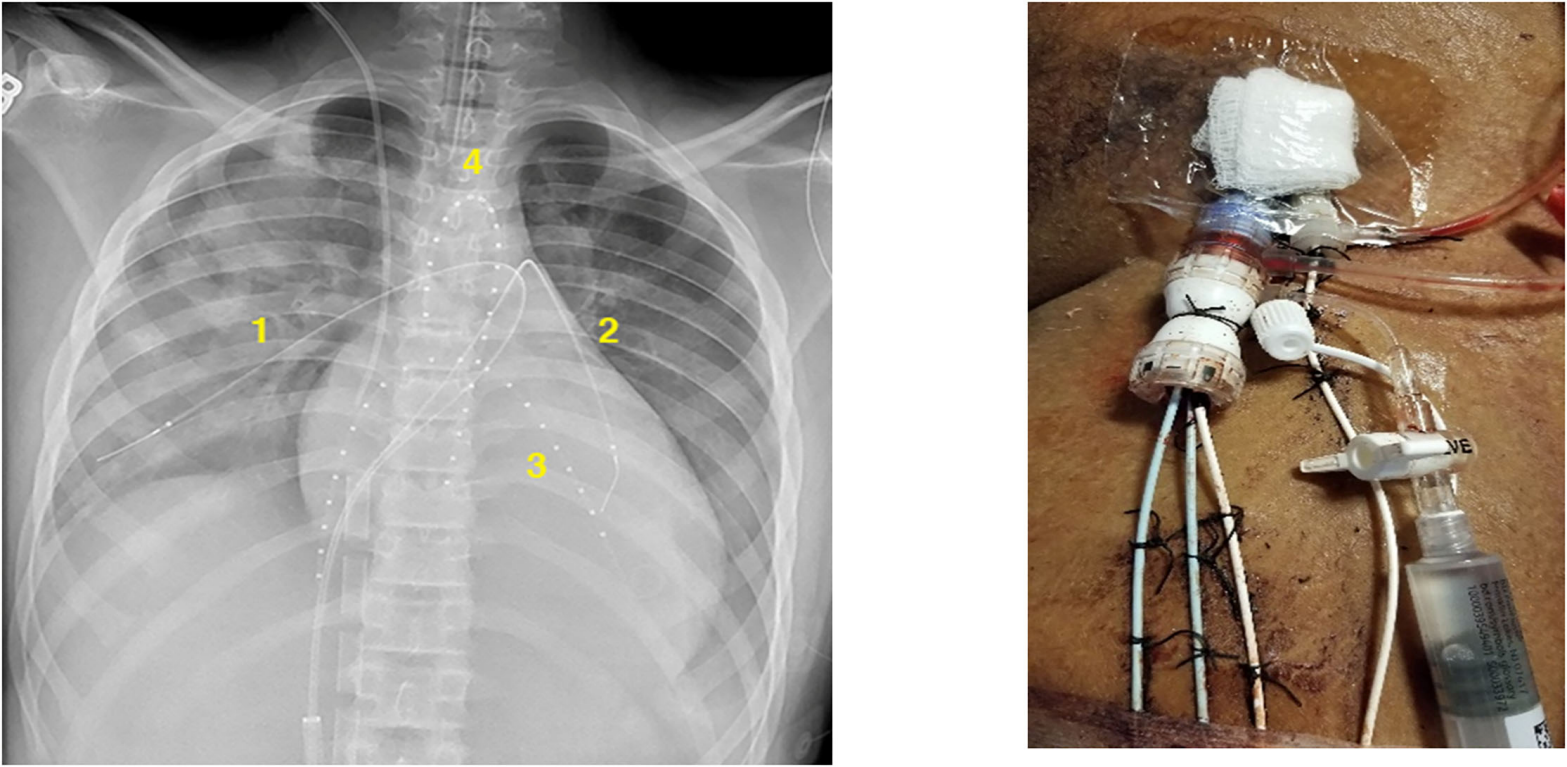
Thrombolysis catheter placement in the right pulmonary artery (1), left pulmonary artery (2), left ventricular cavity (3), and aortic root (4).

CDT was initiated at this time as it was felt that systemic rtPA was ineffective secondary to a recirculation effect due to the proximity of the site of infusion to the ECLS venous drainage cannula in the right atrium. The patient was taken to the cardiac catheterization laboratory and individual catheters were placed at four target sites via the left femoral vessels: one was placed retrograde through a left femoral arterial sheath to the aortic root, one was placed into the apex of the left ventricle via trans-septal approach, and the others were placed in the distal RPA and LPA (Figure 1). A dose of 0.25 mg/h of rtPA with 15 mL/h of fresh frozen plasma (FFP) was infused through each catheter. With the ineffectiveness of systemic thrombolysis likely due to an ECLS recirculation effect, we had significant concern for the lack of blood flow to the thrombus sites and felt that without concurrent exogenous plasminogen supplementation thrombolysis would have been ineffective. The three catheters utilizing a transvenous approach were placed through a single DrySeal sheath (Gore Medical, Flagstaff, AZ) to minimize the need for individual vascular access sites for each catheter. Bivalirudin infusion was continued with an aPTT goal of ~1.5–2 times his baseline (50–60 s). Follow up echocardiogram 24 h after initiation of CDT revealed complete thrombus resolution. CDT was continued for another 12 h and CT angiography confirmed the resolution of all TE. No bleeding complications were noted during his time on ECLS.

The patient was decannulated from VA-ECLS on hospital day (HD) 9. By HD 11, his echocardiogram revealed normal bilateral ventricular systolic function. He remained on therapeutic anticoagulation with bivalirudin until HD 15 when he was transitioned to enoxaparin. An extensive thrombophilia work-up revealed no inherited cause for his hypercoagulability. On HD 15, speech deficiencies were noted and a magnetic resonance imaging (MRI) of his brain revealed no acute abnormalities but did find a chronic punctate lesion in the right cerebellum. After spending 9 days in inpatient rehabilitation, he was discharged home with his family. He was noted only to have mild articulation difficulties that did not impact intelligibility and mild gross motor difficulties (unsteady gait). He was anticoagulated for a total of 12 months with no residual effect noted from his acute illness at the time of anticoagulation cessation.

## Thrombolysis in Children

### Thrombolysis

Although anticoagulation is utilized in the management of acute pediatric TE, anticoagulation alone may not be sufficient to prevent long-term morbidity associated with acute TE events (1). Thrombolysis refers to the use of the exogenous serine proteases tissue plasminogen activator (tPA) and urokinase-type plasminogen activator for more rapid dissolution of thrombus burden (2). Clinical studies for all thrombolytic agents are lacking in children, but studies have shown that tPA may be more efficient at stimulating thrombolysis as it binds preferentially to plasminogen that is fibrin bound (3). Due to its short half-life and the seemingly improved efficiency compared to other agents, rtPA has become the most commonly utilized thrombolytic agent in children (1, 4).

### Methods of Thrombolysis

Thrombolytic therapy has been employed for the management of pediatric TE for decades and the use is increasing (5, 6). A major contributing factor to the rise in pediatric thrombolysis is the increase in pediatric interventional radiologists and cardiologists with an expertise in this therapy (1). Thrombolytic therapy can be administered either systemically or via an endovascular route, including CDT or pharmaco-mechanical thrombolysis. Currently there are no studies comparing the routes of administration in children, which limits the ability to address the relative risk and benefit comparison between systemic and endovascular thrombolysis, but there are case series that suggest CDT may be safer and be more efficacious than systemic thrombolysis (1, 7, 8).

### Thrombolysis Dosing

#### Systemic Thrombolysis

The recommended rtPA dosing for as well as the concomitant use of unfractionated heparin with systemic thrombolysis widely varies (2). Administered treatment regimens include a low dose (0.01–0.06 mg/hg/h) infusion for 6–72 h or a high dose (0.1–0.6 mg/kg/h) for 2–6 h. Laboratory monitoring to assess thrombolytic response and bleeding risk is recommended every 6–12 h and includes a complete blood count, prothrombin time, partial thromboplastin time, fibrinogen, and D-dimer (1, 9). Almost 80% of pediatric patients receiving systemic thrombolysis achieve complete or partial TE resolution, but up to 15% have major bleeding complications (fatal bleeding, hemoglobin drop of at least 2 g/dL in a 24 h time period, any bleed requiring surgical intervention, and specific locations: retroperitoneal, pulmonary, and intracranial) (6, 10). An increased risk for major bleeding in children receiving systemic thrombolysis has been associated with lower fibrinogen activity right after completion of thrombolysis and longer rtPA infusions (11).

#### Endovascular (CDT/Pharmaco-Mechanical) Thrombolysis

Endovascular thrombolysis dosing is typically 0.01–0.03 mg/kg with a maximum dose of 1–2 mg/h. Laboratory monitoring is similar to that of systemic thrombolysis (1). Partial or complete resolution is seen in up to 93% of pediatric patients receiving endovascular thrombolysis (6). While the directed therapy may lower the risk of major bleeding (reported in up to 3% of children who undergo endovascular thrombolysis), it does require more healthcare utilization, including: longer intensive care stays, utilization of interventionalists, and general anesthesia (1).

### Indications for Thrombolysis

Specific indications for thrombolysis in pediatric TE are lacking due to the lack of clinical trials (2). In fact, recent American Society of Hematology guidelines for the management of pediatric venous thromboembolism suggest using anticoagulation alone in acute pediatric VTE and sub-massive pulmonary embolism (PE) over the use of thrombolysis followed by anticoagulation due to the concern that the potential benefit would outweigh the inherent risks (major bleeding—particularly in neonates) in most clinical scenarios (4, 7). In general, thrombolytic therapy is reserved for life-, limb-, and organ-threatening events, including PE with hemodynamic compromise, in centers with access to pediatric interventional radiology or interventional cardiology expertise (4, 7). Yet, with improved laboratory monitoring capabilities, radiographic imaging, and interventional radiology and surgical interventions, the use of thrombolysis in pediatrics has risen in the last 10–20 years and recent guidelines acknowledged that certain patients could benefit from this therapy (1, 7). Thrombolysis is also more likely to be considered in centers with access to interventionalists. Generally, single or potentially two concomitant rtPA CDT infusions may be utilized and there is a report of three distinct simultaneous infusions, but our case, to our knowledge, is the first reported use of four simultaneous rtPA infusions for CDT (12, 13).

### Thrombolysis in Pediatrics

Despite the lack of specific evidence in pediatric populations, there are many reported case series and cohort reports on the use of thrombolysis in pediatric populations that show similar results in efficacy, major bleeding, and TE recurrence rates (Table 1) (14–39). Even though there are many reports on the use of thrombolysis in pediatric patients, the decision to utilize any mechanism of this therapy should be decided on a case-by-case basis (40).

**Table 1:** Summary of case series and case cohorts on thrombolysis in children based on thromboembolism type.

	**References**	**Thrombolysis**	**Site**	**Age range**	**N**	**Result**	**SAE**	**Progressive/** **Recurrent TE**
**DVT**								
	Goldenberg et al. (14)	Systemic/PMT	LE	1–21 y	9	89% (≥90% resolution)	1 pulmonary hemorhage	None
	Golderberg (15)	CDT/MT/PMT	UE and LE	11–19 y	16	88% (≥90% resolution)	1 PE	27%
	Darbari et al. (16)	CDT/MT/PMT	UE and LE	13 d−21 y	34	69% (≥50% resolution)	1 major GI hemorrhage, 2 PRBC	Not reported
	Kukreja et al. (17)	CDT/PMT	UE, SVC, LE, IVC	0–24 m	11	100% (≥50% resolution)	1 PE	None
	Dandoy (18)	CDT/MT/PMT	UE, SVC, LE, IVC	3 m−21 y	41	90% (≥ 50% resolution)	1 major hemorrhage (tracheostomy)	Not reported
	Lungren et al. (19)	CDT/PMT	UE	20 d−17 y	9	100% (100% resolution)	1 PE	None
	Gaballah et al. (20)	CDT/MT/PMT	LE	1–18 y	57	94% (≥50% resolution)	1 major GI hemorrhage	12%
	Cohen et al. (21)	CDT/PMT	LE and IVC	0–19 y	29	35% (100% resolution)	1 major hemorrhage	35%
	Kumar et al. (22)	CDT	UE (Paget-Schroetter)	13–19 y	10	70% (100% resolution)	None	Not reported
	Warad et al. (23)	CDT/MT/PMT	LE (May-Thurner)	8–17 y	7	29% (100% resolution)	None	57%
**PE**								
	Bavare et al. (24)	Systemic/CDT	PE	11–17 y	5	67% (100% resolution)	None	20%
	Pelland-Marcotte et al. (25)	Systemic/CDT	PE	0–18 y	12	Not reported	6 major hemorrhages	33%
	Akam-Venkata et al. (26)	CDT/PMT	PE	12–20 y	9	Not reported	None	Not reported
	Ji et al. (27)	CDT/PMT	PE	6–19 y	9	44% (100% resolution)	1 non-CDT related death	None
	Ross et al. (28)	Systemic/CDT	PE	0–18 y	18	Not Reported	1 extracranial hemorrhage	6%
	Belsky et al. (29)	CDT/PMT	PE	3–21 y	5	80% (100% resolution)	None	None
**CSVT**								
	Mallick et al. (30)	CDT	CSVT	18 m−11 y	4	25% (100% resolution)	1 major retroperitoneal hemorrhage	Not reported
	Mortimer et al. (31)	CDT/MT	CSVT	18 m−16 y	9	“partial”/“good” in all patients	None	None
	Waugh et al. (32)	CDT/PMT	CSVT	9–17 y	6	“Improvement” in 83%	2 ICH hemorrhage	50%
**Abdominal**								
	Koo et al. (33)	CDT/MT/PMT	PVT, SMV, IMV, SV	3 m−17 y	10	77% (100% resolution)	1 hemoperitoneum, 1 hemothorax	10%
**Stroke**								
	Janjua et al. (34)	Systemic	Stroke	1–17 y	46	Not reported	10.9% (DVT, pneumonia)	Not reported
	Rambaud et al. (35)	Systemic/CDT/MT/PMT	Stroke	10–18 y	19	Not reported	None	None
**Multiple sites**								
	Manco-Johnson et al. (36)	Systemic	UE, SVC, LE, IVC, PE, Atrial	6 w−17 y	32	50% (≥90% resolution)	1 PE, 1 death	13%
	Gupta et al. (37)	Systemic/CDT		2 d−18 y	79	65% (100% resolution)	39% with major bleeding requiring PRBC	Not reported
	Wang et al. (38)	Systemic	UE, SVC, LE, IVC, PE, CSVT, RA, LA, LV, Arterial	0–17 y	35	83% (≥90% resolution)	1 embolic stroke	None
	Al-Jazairi et al. (39)	Systemic	UE, SVC, Intracardiac	40 d−13 y	5	40% (100% resolution)	3 major hemorrhages, 1 death	Not reported

*CDT, catheter-directed thrombolysis; CSVT, cerebral sinus venous thrombosis; d, day; DVT, deep vein thrombosis; GI, gastrointestinal; ICH, intracranial hemorrhage; IMV, inferior mesenteric vein; IVC, inferior vena cava; LA, left atrium; LE, lower extremity; LV, left ventricle; m, month; MT, mechanical thrombolysis; PE, pulmonary embolism; PMT, pharmacomechanical thrombolysis; PRBC, packed red blood cells; RA, right atrium; SAE, serious adverse event; SMV, superior mesenteric vein; SVC, superior vena cava; TE, thromboembolism; UE, upper extremity; y, year*.

CHEST recently published consensus recommendations for high-risk and intermediate-risk PE (40). For high-risk PE in adults, there is evidence showing systemic thrombolysis reduces recurrent PE and mortality risk and treatment algorithms for children do recommend systemic thrombolysis in this setting, with consideration of CDT if the facility has staff experienced with this therapy (7). ECLS should also be available in patients with confirmed or suspected high-risk PE in which thrombolysis is being considered (40). For intermediate-risk PE, current adult guidelines recommend against the use of systemic thrombolysis due to the increased risk of major bleeding (41). Yet, there are pediatric case series with more favorable outcomes utilizing CDT in intermediate-risk PE (24, 26, 27, 29). Thus, if thrombolysis is pursued in intermediate-risk PE, CDT may be preferable due to the lower complication rate (40).

More recently, symptomatic IVC and iliofemoral DVT, including those related to May-Thurner anatomy, have been considered indications for thrombolysis. There is evidence to support this decision as it appears to improve function and pain in the short term and it could reduce the risk for the development of post-thrombotic syndrome in the long term (PTS) (14, 15, 18). Similar to the adult data, the combination of CDT and iliac vein stenting followed by anticoagulation is the most common treatment for VTE In the setting of May-Thurner, the evidence suggests that complications using this strategy are low (42). It seems reasonable to consider thrombolysis in pediatric patients with May-Thurner based on the short-term benefits, seemingly low complication rate, and potential long-term functional gains.

In the setting of Paget-Schroetter in adults, there is significant evidence showing the success of thrombolytic therapy and CDT has become the primary method for establishing primary reperfusion (43). The data is much more limited in pediatrics as there is a case series that suggests CDT followed by rib enumeration and anticoagulation is safe and efficacious, but there was no a significant difference in the reported PTS or health related quality-of-life scores between the group that did get thrombolysis and the one that did not (22). There was also no difference in the rate of recurrent DVT. Thus, the need for thrombolysis is still unclear, but could still be considered in certain patients considered to the highest risk for recurrence.

The evidence for the use of thrombolysis in pediatric stroke is also very limited and is generally extrapolated from adult data. There is a recent case series that shows that thrombolysis can be utilized in adolescent stroke for acute revascularization relatively safely and efficaciously (35). Yet, the main multi-institutional trial evaluating the safety and efficacy of thrombolysis in children (TIPS trial) was not able to enroll any patients despite screening 93 patients and confirming 43 with an arterial ischemic stroke (44). Stroke recognition with rapid diagnosis and stroke management strategies were quite varied as the TIPS trial was being organized, but this study did lead to significant increase in acute stroke teams for centers participating in the trial (45). The development of acute pediatric stroke alert teams may fill in existing knowledge gaps, but, until then, current data only suggests that decision for acute revascularization with thrombolytic therapy in pediatric patients should involve multidisciplinary collaboration and be made on a case-by-case basis (35).

#### Extracorporeal Life Support

ECLS is a technique that maintains gas exchange, tissue oxygenation, and cardiac output in patients with temporary, reversible cardiac and respiratory failure (46). Since the 1990s, ECLS has been increasingly utilized in pediatric intensive care units (47). As anticoagulation is required for pediatric ECLS, major difficulty in the management is balancing the risk for both hemorrhage and thrombosis. TE in children can lead to the need for ECLS and TE can occur while on ECLS, and the management of these situations can be quite challenging. There are clinical situations, including the one presented in our case, where the benefits of thrombolysis outweigh the risk and the use of CDT in pediatric ECLS has been safe and effective (48–51).

There are also reports of neonates and infants receiving thrombolysis while on ECLS: a 4-day-old with acute respiratory failure on ECLS that developed a RA thrombus, a 4-day-old with an acute MI and cardiogenic shock, a 12-day-old with respiratory failure in the setting of a congenital diaphragmatic hernia that developed an aortic thrombus to the level of the kidneys in the setting of a umbilical arterial catheter on ECLS, and a 7-month-old with a bidirectional Glenn shunt thrombosis prior to ECLS (13, 52–54). Generally, CDT is preferred in neonates compared to systemic TPA due to the lower risk for bleeding, which is only amplified on ECLS (55). There is also a special consideration in neonates, namely developmental hemostasis and decreased plasminogen levels compared to adults. Some of the reported neonatal cases required a concomitant FFP infusion to ensure adequate thrombolysis was achieved. This principle was employed in our case as there was concern that the stagnant flow within the central vasculature would have limited plasminogen availability for successful thrombolysis. Despite the reported successful utilization of thrombolysis on ECLS in children, the therapy should only be considered in certain pediatric patients after consultation with the family and providers trained in thrombolysis with a full understanding of the risks and benefits (55).

#### COVID-19 and MIS-C

Acute COVID-19 infections have been associated with hypercoagulability and thromboembolic complications (56). There is a report of an adolescent successfully utilizing dual CDT for segmental and subsegmental PE in the setting of acute COVID-19 as well as a 21-year-old with recurrent PE undergoing successful CDT for bilateral PE (12, 57). Yet, in adolescents and children, MIS-C has been shown to have a higher TE incidence than acute COVID-19 infections (58). And while CDT has been successfully utilized in the setting of acute COVID-19, it appears that our case may represent the first reported successful use of thrombolysis in the setting of MIS-C (49).

Thrombolysis has been utilized anecdotally for the management of acute COVID-19 infections with TE complications, but the evidence is limited (49, 59). The logistical challenges that thrombolysis present in a patient with acute COVID-19, including the complexity of transporting patients with COVID-19 due to the potential nosocomial spread of the virus, may limit the ability to utilize this therapy (60). Despite this limited evidence and the logistical challenges, the National PERT Consortium has stated that the indications for thrombolysis for acute COVID-19 associated pulmonary embolism remain unchanged (61). Extrapolating from this recommendation, it seems that thrombolytic therapy can still be considered in the setting of an acute COVID-19 infection understanding there may be instances where the risk is too significant to proceed.

Heparin resistance, similar to our patient, in acute COVID-19 has been described, mostly in adults admitted to intensive care units (56, 62). Typically, patients receiving CDT and some receiving systemic thrombolysis will also receive prophylactic dosing of unfractionated heparin. Yet, there are instances where heparin may not be effective, and an alternative anticoagulant must be considered. There have been two reported cases in children of the successful use of bivalirudin with CDT. The first is a 2-year old with a history of a mechanical heart valve that presented in shock and developed significant thrombotic burden while on high doses of UFH (63). The other is a teenager with known antithrombin deficiency with inadequate anticoagulation on low molecular weight heparin (64). Our case supplements the fact that bivalirudin can be utilized safely and effectively in heparin resistant children during thrombolysis.

## Conclusion

As the incidence of pediatric TE has increased, the utilization of thrombolysis has as well. While there is some evidence of the acute and chronic benefits of thrombolysis in pediatric patients, most of the data is extrapolated from adult studies. Thrombolysis can be utilized safely in children, but it requires multidisciplinary collaboration with experienced providers from numerous specialties (hematology, interventional radiology, interventional cardiology and/or critical care) as well as close laboratory monitoring. Without specific evidence from randomized trials in children on the risks and benefits of thrombolysis, providers must be vigilant in the patients selected for the therapy and ensure appropriate monitoring is undertaken to ensure the most optimal outcomes.

## Author Contributions

GW and HV contributed to the conception and design of the manuscript. GW wrote the first draft of the manuscript. All authors contributed to manuscript revision, read, and approved the submitted version.

## Conflict of Interest

The authors declare that the research was conducted in the absence of any commercial or financial relationships that could be construed as a potential conflict of interest.

## Publisher's Note

All claims expressed in this article are solely those of the authors and do not necessarily represent those of their affiliated organizations, or those of the publisher, the editors and the reviewers. Any product that may be evaluated in this article, or claim that may be made by its manufacturer, is not guaranteed or endorsed by the publisher.
